# Group-based healthy lifestyle workplace interventions for shift workers: a systematic review

**DOI:** 10.5271/sjweh.3763

**Published:** 2018-09-09

**Authors:** Evangelia Demou, Alice MacLean, Lismy J Cheripelli, Kate Hunt, Cindy M Gray

**Affiliations:** 1 MRC/CSO Social and Public Health Sciences Unit, Institute of Health and Wellbeing, University of Glasgow, Glasgow, G2 3AX, UK; 2 School of Social and Political Sciences, Institute of Health and Wellbeing, University of Glasgow, Glasgow, G12 8RS, UK; 3 Institute for Social Marketing, Faculty of Health Sciences and Sport, University of Stirling, Stirling, FK9 4LA, UK

**Keywords:** group-based, healthy eating, healthy lifestyle, intervention, physical activity, review, sedentary behavior, shift work, shift worker, systematic review, weight, workplace intervention

## Abstract

**Objective:**

Shift work is a risk factor for many chronic diseases and has been associated with unhealthy lifestyle behaviors. Workplaces have great potential for promoting and supporting behavior change. We conducted a systematic review of group-based lifestyle workplace interventions for shift workers to (i) identify adaptations and intervention components that accommodate shift working and (ii) assess their impact on weight, physical activity, sedentary behavior and healthy eating.

**Methods:**

A systematic search was conducted in Scopus, Web of Knowledge, EBSCO and Ovid databases. Using pre-established criteria, independent pairs of researchers conducted the study selection, quality appraisal and data extraction.

**Results:**

In total, 22 studies on group-based workplace interventions for shift workers were included. Many demonstrated organizational level adaptations, such as flexible delivery times and paying employees’ time for their involvement. Delivery locations near the workplace and management support were other key features. Common intervention components included competitive group activities, individualized goal setting, self-monitoring and feedback, staff involvement in intervention delivery, and incentives. There was moderate evidence for effectiveness on weight and physical activity outcomes, but insufficient evidence for healthy eating outcomes. No interventions focusing on sedentary behavior among shift workers were found.

**Conclusion:**

Current evidence demonstrates that group-based workplace interventions can be effective for supporting shift workers to lose weight and increase physical activity, while further research is needed to change healthy eating and sedentary behaviors. Our findings offer decision support on organizational-level adaptations and intervention components that are important to make interventions that promote healthy lifestyles more accessible to shift workers.

Healthcare expenditure has risen continually over recent decades (1–5), and a significant proportion of these costs can be attributed to chronic health conditions such as diabetes, cardiovascular disease and asthma (6). Chronic diseases are the leading causes of death and disability worldwide and affect all socioeconomic groups. Poor lifestyles, including an unhealthy diet and physical inactivity, are associated with being overweight or obese as well as many chronic health conditions, reduced physical functioning, functional impairment and early exit from the labor market onto disability pensions (6–8). These factors can hinder individuals’ opportunities for extending their healthy working lives, and present employers with challenges such as higher sickness absence rates, reduced productivity, and premature loss of valued employees (9).

Unhealthy lifestyles are amenable to change, but achieving sustained behavioral change is difficult. Workplaces, as physical and social environments, have great potential for facilitating more positive lifestyle choices. Over 75% of working age people are in paid work and spend much of their waking time working (10). Employers have legislative responsibilities for health and safety, and there is a strong business case for investing in appropriately-scaled initiatives to promote healthy lifestyles in the workplace, reduce employee turnover, increase productivity and employee engagement, and contribute to corporate social responsibility for the UK’s ageing workforce (10, 11).

Workplace lifestyle interventions have been shown to improve employee health, increase productivity and be cost effective (6), and many are tailored to suit the specific operational and organizational requirements of different workforces (12–21). Group-based workplace interventions offer the advantage of peer support and cost-effectiveness, and are often the preferred option for healthy lifestyle initiatives in the workplace (22–24). There is evidence of the positive impact of workplace programs on health behaviors (25): increases in self-reported physical activity have been demonstrated, particularly in workplace interventions targeting physical activity (including walking) as opposed to general lifestyle change (26, 27). Workplace dietary interventions have also been shown to improve eating behaviors, for example increased fruit and vegetable intake and decreased fat intake (28). Other positive outcomes include: improvements in psychosocial health, quality of life and emotional well-being (29); and reduced presenteeism (29–31), absenteeism (31) and sickness absence (32).

## Shift work and health risks

Recent reports highlight the ongoing discussion as to whether shift work should be classified as an occupational hazard (33). Shift work has been shown to be a risk factor for many chronic diseases (33–38), and links between shift work and weight gain, type-2 diabetes, coronary heart disease, stroke and cancer have been demonstrated (35). Shift work has also been associated with physical inactivity and poor diet (39). A recent study of the dietary characteristics of shift workers found that that while diet quality does not differ between shift workers and those working regular hours, doing night shifts was associated with higher energy intake (36). Fujishiro et al (34) examined the cumulative exposure to rotating night shifts among more than 50 000 women from the Nurses’ Health Study II, and demonstrated an independent contribution of night shift work to weight gain. The development of workplace lifestyle interventions specifically for shift workers is an emerging field (33). Such interventions require an approach that considers and accommodates not only the worker and operational characteristics, but also the organizational determinants that can act as barriers or enablers to successful implementation (33, 40). This study aims to identify the existing evidence for group-based interventions delivered within workplace settings to help shift workers lose weight, increase physical activity, improve healthy eating, or reduce sedentary time.

## Methods

### Inclusion criteria

We included any studies and study protocols that described group-based interventions delivered in work-place settings, specifically targeting shift workers in the public or private sectors. Group-based interventions were defined as any interventions that delivered the intervention or specific components of the intervention to groups of employees rather than on an individual basis. For instance, this could include group educational seminars, team-based physical activity challenges and group physical activity training sessions to name a few. The intervention had to target at least one of the following: weight loss; physical activity; dietary improvement; and/or reduced sedentary behavior. The target population had to be aged 18–70 years. Outcomes of interest were weight, physical activity, diet, and/or sedentary time.

### Exclusion criteria

Interventions delivered to the self-employed or employees working in small- to medium-sized enterprises were not included. Studies were excluded if there was no mention of shift work and/or interventions were delivered in a work setting that the research team agreed was not likely to involve shift working. Additionally, studies detailing interventions that were purely web-based were excluded from the review, as were studies with participants who had undergone weight loss surgery.

### Search strategy

To devise our search strategy, the four main concepts relevant to the review were identified: (i) lifestyle; (ii) interventions; (iii) shift workers; and (iv) setting. A search term list was then developed for each concept, which included free-text terms and comprehensive controlled vocabulary items. We included both UK and US spellings and used truncation to capture plural and singular forms of words. We also used search techniques such as Boolean and proximity operators and phrase searching. We applied limits to year of publication from 2000 to the date of search (12^th^ April 2018). The other limits applied were: human, adult and English language.

Our final search strategy was: [(Weight W/2 (Manage* OR Los* OR Reduc*)) OR ((Physical OR Exercis*) AND (Capacity OR Perform* OR Train* OR Effort* OR Exert*)) OR (Healthy W/2 (Diet* OR Nutrit* OR Eat* OR Food)) OR (Lifestyle)] AND [(Group AND (Program* OR Interven*))] AND [((Employee* OR Staff* OR Worker* OR Personnel* OR Workforce)) OR (Shift* W/2 Work*) AND ((Job OR Work) W/2 (Place* OR Site*))]. The search strategy was adapted to the specific requirement of each different database used.

**Figure 1:**
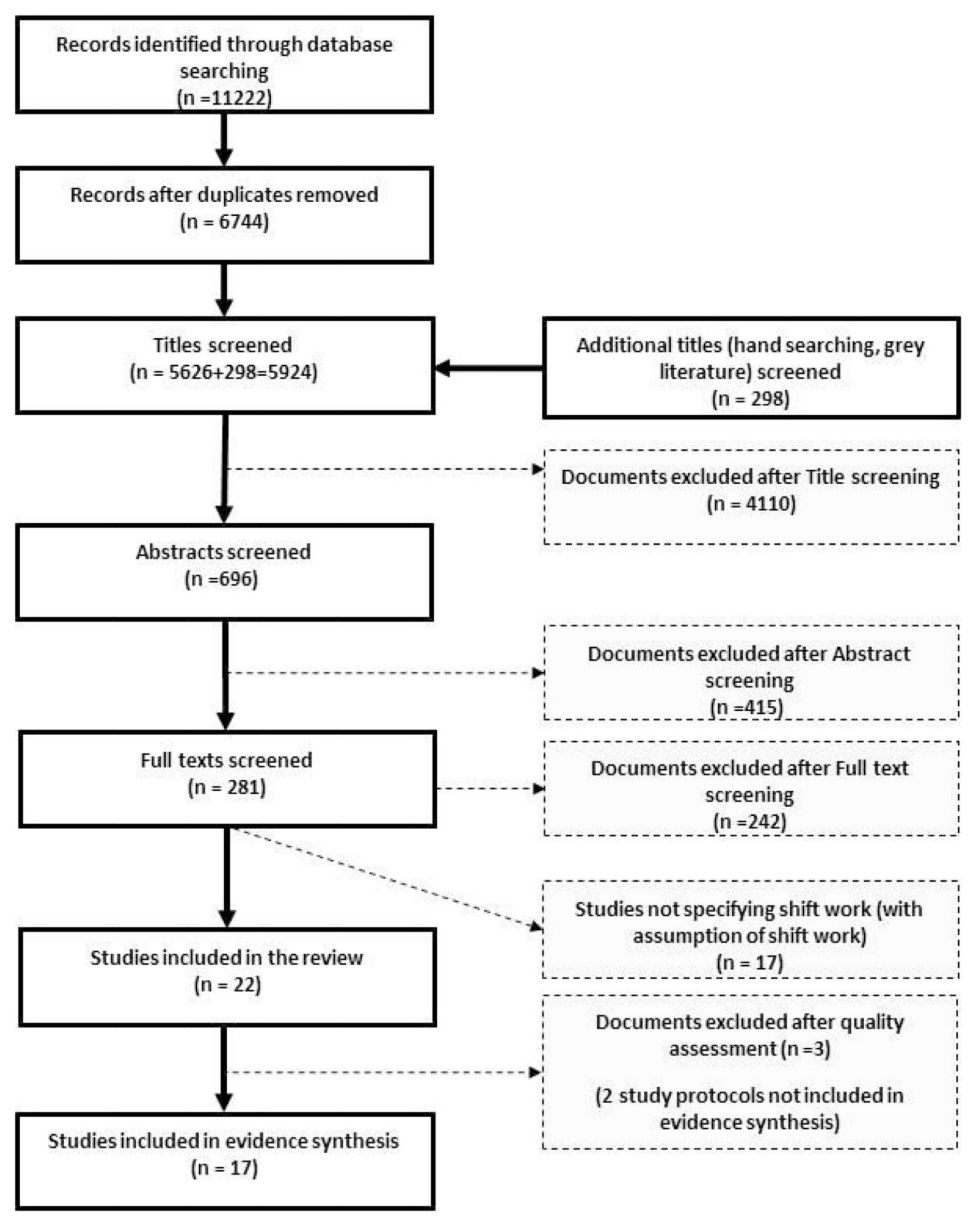
Flow chart of the selection process of included studies

To be as inclusive as possible, we consulted databases across the medical, public health, and social science disciplines: Scopus, Web of Knowledge, EBSCO (CINAHL, PsychInfo, Francis, and SocIndex), Ovid (EMBASE and Medline). All authors participated in screening the titles, abstracts and full papers using the inclusion criteria defined in the previous paragraphs. After excluding duplicates, a total of 5626 citations were obtained from the electronic search (figure 1). A further 298 citations were identified through hand-searching reference lists and the grey literature, leading to 5924 citations for screening. Each unique title was independently assessed by two reviewers, and where they did not agree, a third reviewer was consulted in order to reach a consensus.

After title screening, two reviewers independently reviewed 696 abstracts for eligibility. Of these, 281 publications were identified for full-text review. Two reviewers assessed all full texts, and a third reviewer independently read 10% for quality control. Any discrepancies were resolved by discussion. After full-text screening, 242 papers were excluded, and reasons for exclusion were noted. A further 17 papers were subsequently excluded as shift work was not specifically evidenced.

### Data extraction

In order to develop the final data extraction table, members of the research team used a pilot version to independently extract data from a sub-sample (16%) of the included papers. The final version contained seven fields: first author and publication year; country; study design; workplace setting; study participants/sample size, intervention aim; and intervention description (including components).

### Quality assessment

Two reviewers independently assessed the methodological quality and sources of potential bias of all included studies using the Consolidated Standards of Reporting Trials (CONSORT) Statement (41) for randomized studies (25 items) and the Transparent Reporting of Evaluations with Nonrandomized Designs (TREND) Statement (42) (22 items) for non-randomized studies. Both quality assessment tools were used to systematically examine and appraise: title and abstract; scientific background and introduction; methods; results; and discussion. All items were rated as 1 when the condition was satisfied, 0.5 when it was partially satisfied and 0 if the condition was not met. The included studies were then classified as high, moderate or low quality if their final assessment score was >80%, 60–79%, and <60% of the maximum possible score (42), respectively. The two reviewers discussed any disagreements and, if necessary, resolved with reference to a third reviewer. Detail results of the quality assessment of all included studies can be found in the supplementary material (tables S1 and S2, www.sjweh.fi/show_abstract.php?abstract_id=3715).

Following quality assessment, three papers were rated as low-quality (43–45); these were included in the descriptive analyses, but excluded from the evidence synthesis (see below). Reasons for low quality included: lack of detail on the method used to generate the random allocation sequence or on the type of randomization; no information on the mechanism used to implement the random allocation sequence; and the absence of eligibility criteria for participants. Five study protocols were also excluded from the evidence synthesis. Seventeen studies of high or moderate methodological quality were included in the evidence synthesis (figure 1 and supplementary tables S1 and S2).

### Analysis

To identify themes emerging from the review and key intervention components, a narrative synthesis approach was undertaken using three distinct steps: collating, summarizing, and reporting the results (46). The information was collated in tables, and the main findings summarized and reported by outcome of interest. This involved an iterative process, examining the evidence for intervention components that may have influenced the outcomes. Study protocols were included in this stage.

In order to assess the effectiveness of group-based workplace interventions, we performed an evidence synthesis based on the quality assessment rating and the significance or non-significance in relation to the outcomes of interest (weight, healthy eating, physical activity, sedentary behavior) and other relevant outcomes (objective and self-reported health, sickness absence and other work-related outcomes). The criteria used for the evidence synthesis were: "strong evidence" – consistent results (in terms of statistical significance between ≥2 high quality studies; "moderate evidence" – consistent results between ≥1 high quality and ≥1 intermediate quality study, or between ≥2 intermediate quality studies; "insufficient evidence" – identification of a single study or inconsistent results across studies; and "evidence of no association" – consistent results of a non-association in ≥2 studies (47, 48). Based on the definitions of Stennstra et al (48), a significant effect in one study and a non-significant effect in another were considered consistent findings, while a negative effect in one study and a positive effect in another were considered inconsistent findings.

## Results

### Characteristics of included studies

In total 22 studies were included in our review, and 17 of these in the evidence synthesis (figure 1). The main reasons for exclusion were: not a group-based intervention; not adapted for shift work; email, postal, purely web-based, environmental or individual (ie, one-to-one delivery) intervention; not a workplace setting; and target health behaviors and outcomes (eg, smoking, alcohol consumption) beyond the scope of this review.

The included studies were conducted over four continents (table 1), with 8 from North America [7 USA (44, 49–54), 1 Canada (43)], 8 from Europe [2 Denmark (55, 56), 1 UK (57), 1 Netherlands (58), 1 Finland (59), 2 Norway (60, 61), 1 Ireland (62, 63)], 5 from Australia (12, 45, 64–66) and 1 from South America [Brazil (67)]. There was a wide range of workplace settings including hospitals and care/nursing homes, manufacturing, fire and prison services, hospitality, casinos, transportation and other public and private sector organizations (table 1). The most common study design was the randomized controlled trial (RCT) (11 studies), followed by pre-post intervention (10 studies), and one study had a quasi-experimental design. The review includes 9725 participants in total [smallest study size: 33 participants (66), largest study size: 4536 participants (51)]. The majority of the included interventions had a focus on increasing physical activity (19/22; 86%) either alone or as part of broader interventions; 14 studies (64%) had a focus on improving diet (only 2 targeted diet alone), and 6/19 studies (32%) were holistic interventions with a primary focus on weight loss. Only 1 of the included studies reported sedentary behavior outcomes (self-reported) (45).

### Intervention delivery

We found a range of delivery formats for workplace lifestyle group-based interventions for shift workers; from programs based around face-to-face information sessions (50, 64) or guided physical activity sessions (57, 60, 61, 68), to more composite programs that included educational lectures, supervised or structured PA sessions, guidance for individual PA activities and/or counselling sessions (12, 43–45, 49, 51–56, 59, 62, 63, 65–67). Some organizational level adaptations were identified as being important to make the interventions more accessible to shift workers. These included flexibility in timing of delivery (12, 43, 45, 53, 54, 60–63, 68), such as scheduling activities immediately before, after, and/or during shifts (51), or ensuring that activities were offered at different times to cover employees on all shifts (45, 50, 62, 63). Other companies gave shift workers time off work for participation (53) or paid for employee time both to deliver (51) and participate (52) in the intervention. Another common measure was to ensure that physical activity sessions were held as near as possible to the workplace (52, 66). Management support (45, 50–53, 57) and encouragement (12, 50–52) for employees to join and continue to take part in the intervention were also used to support program delivery, and in two cases both management and shift working employees took part in co-production activities during intervention development (44, 45).

### Intervention components

The interventions all included multiple components (table 1) often operating at different levels, including individual and environmental. A number of components featured in a large number of the interventions. These included competitive group activities (44, 45, 49, 51, 52, 57, 64–66), behavioral modification strategies such as individualized goal setting, motivation techniques, and feedback (12, 43, 45, 49, 51, 52, 54, 55, 57, 59, 64, 67), a leader or "go-to" person as a point of contact (43, 49, 51, 53, 57, 64, 67, 68), and incentives (eg, gift vouchers, coupons) (12, 49, 52, 53, 64–66). Peer support systems were included by utilizing peer champions (49), exemplar behavior from other staff or management (44), group leaders (51), and team competitions (45, 52, 64).

A wide range of resources were used to support intervention delivery including leaflets, fitness, trackers, personal trainer, dedicated webpages and counselling sessions (12, 43–45, 49, 51–55, 57, 59–68).

### Components of interventions targeting weight

Interventions focusing on weight loss (43, 51, 53, 58, 64, 65) included group education sessions, sometimes combined with one-to-one information or counselling sessions or individualized support and feedback. A range of resources was used, including dedicated websites to complement workplace delivery, handbooks, pedometers, diet logbooks and healthy eating resources or supplies (eg, provision of free fruit). A strong emphasis was placed on the importance of group activities and peer support, and team competition was often included. Incentives in the form of financial prizes for teams (64, 65) or individuals (53) were also used. Environmental components included healthy options and portion sizes in the cafeteria (51).

### Components of interventions targeting physical activity

The types of physical activity exercises offered were wide ranging (eg, aerobics, walking sessions, weight training, dancing, step challenges). The use of free resources, including pedometers/fitness trackers (49, 52, 53, 59, 61, 64–67), and feedback directly from an instructor, or via or printed material (43, 45, 49, 51, 52, 56, 59), was a key feature. The interventions often relied on team-based competitions to motivate employees to become more active (12, 44, 49, 52, 57). It was recognized that individualized components and tailoring for physical fitness levels were necessary for effective engagement (12, 43, 49, 52, 54, 55, 57, 59, 67).

### Components of interventions targeting healthy eating

The components of interventions with a major focus on dietary improvement (45, 50–52, 62, 63, 65, 66) were often highly similar to the weight loss and physical activity interventions described above. These included free access to health clubs, personal training, food logs, cookbooks and healthy eating supplies (51, 52, 62, 63, 65, 66). Environmental changes included free coupons for healthy meals at the workplace cafeteria, changes in the price for healthy foods or the establishment of "healthy eating chat tables" at the cafeteria (52, 62, 63), and the provision of healthy options and smaller portion sizes (45, 51, 62, 63). One of the interventions provided weekly educational classes delivered by a registered dietician (50), and incorporated an analysis of participants’ health beliefs, nutrition knowledge and dietary behaviors prior to commencing the intervention (50), while another intervention combined both nutrition education sessions with environmental changes at the workplace cafeteria (62, 63).

### Components of interventions targeting sedentary behavior

There were no studies focusing on changing sedentary behavior. However, one study targeting physical activity in truck drivers reported a change in the number of truck drivers sitting for >9 hours each day at work (self-reported) after implementing different interventions or combinations of interventions, including displaying healthy eating posters, supplying free fruit, promoting online resource, group educational sessions or step challenge (45).

### Intervention effectiveness

Evidence synthesis on the effectiveness of the interventions on the reported primary outcomes (as well as other outcomes of interest, eg, health, sickness absence, work ability) was performed on the 17 studies rated as moderate or high quality following the quality assessment. Table 2 indicates that there is moderate evidence for improvements in weight and physical activity, and insufficient evidence for improvements in healthy eating (see also supplementary table S3, www.sjweh.fi/show_abstract.php?abstract_id=3715). Five of the nine studies reporting weight loss outcomes showed positive and significant impacts (52–54, 64, 67); three studies showed no significant difference between intervention and control groups (12, 49, 61, 62), one showed inconsistent results between the intervention and the control arms (62), and one moderate quality study demonstrated a modest negative impact (57). Physical activity had four high or moderate quality studies reporting significant positive impacts (59, 64, 65, 67), three studies reporting a non-significant positive change or inconsistent results (49, 57, 68) and one study reporting positive self-reported change in physical activity levels without indicating if the change was significant or not (66). There were seven studies targeting healthy eating (49, 50, 62, 64–66, 68, 69), only two studies- one high and one moderate quality- reported significant positive impacts (66, 68), whereas five studies reported non-significant or inconsistent results (49, 50, 62–65).

All studies examined a range of health and wellbeing indicators, both objectively-measured (eg, blood pressure, resting heart rate, body fat, fasting lipids, VO_2_ max) and self-reported (eg, perceived health status, self-reported mental health, work ability). Objective and subjective health measures all had comparable numbers of studies reporting either significant positive impacts or non-significant, inconsistent or significant negative results (12, 49, 52, 56, 57, 59–61, 64–66, 68–71). Moderate evidence was available for improvement in some work outcomes: work ability (56, 68–71) and need-for-recovery (59). However, there was no evidence of any impact on sickness absence (56, 60, 69–71) (table 2, supplementary table S3). Heterogeneity meant that it was not possible to assess strength of effect, conduct a meta-analysis, or assess the effectiveness of specific intervention components on our target behaviors.

## Discussion

Group-based workplace interventions to promote weight loss, physical activity and healthy eating behaviors in shift workers require a number of adaptations at the organizational level, including flexible delivery, proximity of intervention sites to the workplace, and management support and encouragement. The flexibility in delivery that was demonstrated reflected the complexity of intervening in workplaces via group-based interventions to improve shift workers’ health behaviors and especially adapting to the specific challenges associated with differing work patterns. The interventions included in this review often targeted more than one of the outcomes of interest (sometimes with other outcomes) and had many components. Competitive group activities, behavioral modification strategies, such as individualized goal setting and feedback, and incentives were key components that featured widely. The results demonstrate moderate evidence of the effectiveness of group-based workplace interventions on weight and physical activity, but insufficient evidence for healthy eating. Moderate evidence was also demonstrated for health and work-related outcomes, but no significant impact on sickness absence was observed.

### Research findings in context with previous studies

Previous research has shown that workplace interventions at the organizational level alone have modest effects on lifestyle behavior (72–74) and that the best evidence for effectiveness is from multi-component interventions that work across different levels (23, 74–77). This was also evident in the studies included in this review: many were multicomponent and included a number of adaptations to reflect shift working patterns and constraints in order to support, promote and implement the interventions. These adaptations and components ranged from changes to the cafeteria environment (45, 51, 62, 63), free resources and access to facilities (45, 49, 52, 53, 57, 59, 61, 64–66, 68), to flexible delivery in the workplace to ensure maximum reach (12, 43–45, 49, 50, 53, 54, 57, 60–63, 66–68).

Significantly positive impacts on diet have previously been observed in interventions delivered in the workplace during work-time, and those that involved staff in delivery and were multicomponent (78, 79). Similarly, the interventions tailored for shift workers included in this review were all either delivered at the workplace, or very close by. Staff involvement was often demonstrated by having peer champions (49), staff or management role models (44), and group leaders (51). Examples of changes at an organizational level were evident in all interventions, including management involvement and support (12, 44, 45, 50–53, 57, 62, 63).

Another review on the general working population suggests that workplace health promotion can improve health outcomes and productivity (80). Goetzel et al’s (80) evidence synthesis on the impact of workplace health promotion interventions on health outcomes showed insufficient evidence overall but did include a number of individual studies demonstrating positive impacts. The studies included in our review demonstrate similar results for physical and mental health outcomes, with a number of studies demonstrating significant positive impacts (12, 49, 52, 54, 60). While we did not assess productivity per se, our review addressed productivity-related outcomes, including sickness absence and work ability (56, 59, 60, 69–71). Brox et al (60) demonstrated an increase in sickness absence in the intervention group, but a non-significant difference in self-certified sickness absence. While Jakobsen et al (56, 69–71) demonstrated a significant increase in self-reported sickness absence in the last year as measured by one item of the work ability index. Pohjonen et al (59) showed no change in work ability. Jakobsen et al (56, 69–71) reported a small to moderate significant effect in work ability, but no changes in other work ability measures (eg, work disability, influence at work).

### Study strengths

A strength of this systematic review is that it included a comprehensive scrutiny of databases covering the medical, public health, and social science literatures. It also covers a large population (total N=9725), from ten countries and four continents. Additionally, it encompasses a breadth of workplaces, including hospitals and care/nursing homes, manufacturing, fire and prison services, hospitality, casinos, transport, and other public and private sectors, each with unique opportunities and often significant challenges for intervention development and delivery, and included sectors (eg, hospital, fire service, care home) which are recognized to have high levels of work-related health problems and sickness absence (81).

### Study limitations

While the range of targeted behaviors and other reported outcomes, workplaces covered and intervention components included in this review is a strength, at the same time the heterogeneity of the included studies and intervention components do not allow for a meta-analysis, or assessment of the effectiveness of specific intervention components on our target behaviors. While the majority of identified studies were RCT, which are regarded as being methodologically robust, in the final evidence synthesis only seven RCT were included. Most of the studies were classified as moderate quality; only three (all RCT) were high quality. The pre/post design of a number of the studies makes it difficult to draw conclusions about causal relationships. Other limitations include the variability, validity and reliability of the reporting of shift work. As only papers that explicitly made some mention of shift work, however tangentially, were included in this review, it is possible that we could have inadvertently excluded other high quality studies, for example where interventions were aimed at an entire workforce, and not only shift workers.

### Implications for policy and practice

Research into the development and implementation of interventions tailored specifically for shift workers is a new and evolving field with many evidence gaps. Papantoniou et al (33), highlight the increasing evidence that shift work increases the risk of major chronic diseases, draw attention to the large proportion of the current workforce exposed to shift work, and call for more workplace interventions addressing health-related outcomes among shift workers. A recent study on the barriers and facilitators to a healthier lifestyle and the impact the working environment can have on shift workers found that the workplace environment was key in assisting shift workers to adopt and lead healthier lifestyles (82). Discussions are ongoing about whether to classify shift work as a workplace hazard qualifying for compensation (33). Denmark already considers breast cancer an occupational disease in shift workers, and compensates women with >20 years of night work who develop breast cancer (33).

Interventions for chronic disease risk reduction and prevention in shift workers require novel approaches to reflect the constraints of shift working. This review suggests a number of adaptations, including flexibility in timing of delivery (49–51, 53, 54, 56, 62, 63), allowing time off work for participation (53) and paying for employee time for intervention delivery and participation (51), that should be considered in developing future workplace healthy lifestyle interventions for shift workers. However, although there is some evidence in relation to weight loss and physical activity, more research is needed in order to maximize impact on lifestyle (including sedentary behavior), health and work-related outcomes.

### Concluding remarks

Workplaces, as physical and social settings, have great potential for promoting health and wellbeing (6, 12–21, 82). Shift work has been associated with unhealthy lifestyle behaviors, which can contribute to increased risk for disease. Our findings suggest that workplace healthy lifestyle interventions with a group-based element can be implemented for shift workers by ensuring flexible delivery modes and organizational level adaptations, and can be effective in promoting weight loss and physical activity. This review can inform the development and implementation of future workplace interventions for shift workers to ensure that this specific workforce population can benefit from their workplace environments by promoting behaviors that protect against chronic diseases.

## Supplementary Material

Please note that there is additional material available belonging to this article on the Scandinavian Journal of Work, Environment & Health-website.

Supplementary Information

## Figures and Tables

**Table 1:** Descriptive table of included studies. [RCT=randomized controlled trials; PA=physical activity.]

Study ID	Country	Design	Setting	Participants/Sample size	Target outcomes	Intervention description
Morgan et al (64)	Australia	RCT	Aluminum smelter plant	Employees: Total N=110 Intervention: N=65 Control: N=45 Gender: Male 100% Age: 44.4±8.6yrs	Weight, PA, Diet	**Intervention delivery** Single face-to-face information session **Intervention components** A 3-month weight loss program consisting of: ▪ Information session: One face-to-face session (75 min): 60 min of education session ▪ Study website: 15 min technical orientation about the free weight loss website; weekly weight recording for 4 weeks, then fortnightly the next month, and for 1 week in the third month; 7 individualized feedback documents via email; supportive weekly email answers from researchers ▪ Resources: weight-loss handbook, website user guide, yamaxsw200 pedometer ▪ Group-based financial incentives at two time points: An $AU50 gift voucher per person for a local sporting equipment store for the groups with the highest mean percentage weight loss after 1 month and at program end
Strijk et al (58,68)	The Netherlands	RCT and study protocol for vital@Work RCT	Dutch academic hospital	Employees: Total N=730 Intervention: N=367 Control: N=363 Gender: Women (%) Intervention: 74.7% Control: 76.3% Age: Intervention: 52.5±4.8yrs Control: 52.3±4.9yrs	PA, Diet	**Intervention delivery** ▪ Intervention modified to fit within a common working day by choosing adequate time schedules for the provided yoga and guided workout group sessions ▪ Guided group sessions provided in two time blocks on all working days: 1) during lunchtime (3 sessions), and 2) after working hours (3 sessions) ▪ Guided group sessions conducted near the worksite (max. 5-10 min walk) **Intervention components** A 6-month lifestyle intervention consisting of: ▪ Personal Vitality Coach (PVC) visits: Three individual visits to a Personal Vitality Coach ▪ Vitality Exercise Program (VEP): Weekly guided yoga group session; weekly guided aerobic workout group session; weekly unsupervised aerobic exercise session (45 min at similar intensity as the guided workout sessions) ▪ Free fruit provision at group sessions
Pohjonen & Ranta (59)	Finland	RCT	The Social Services Department of the City of Helsinki.	Female home care aides (Total N=57) Intervention: N=50 Control: N=37 Age: 41.8±10.4yrs Sex (%)d	PA	**Intervention delivery** ▪ Physical exercise program conducted during work hours; within participants’ own work units ▪ Facilities were near the worksite **Intervention components** A 9-month PA intervention consisting of: ▪ A 2 hour orientation and motivation session on physical fitness offered prior to exercise sessions ▪ Two lectures on leisure-time PA and effective exercise ▪ Supervised exercise (1-hr twice a week): Aerobics (games, aerobic dancing, step aerobics, and gymnastics), Muscular fitness ▪ Heart rate monitors used over entire shift ▪ Printed personal feedback and counselling
Oldervoll et al (61)	Norway	RCT	The University Hospital in Trondheim, Norway	Employees: Total N=65 Endurance (ET): N=22 Strength promotion (SP): N=24 Waiting list group (CON): N=19 Gender: 100% female Age: ET: 43.9±8.8yrs SP: 42.6±6.0yrs CON: 42.2±6.0yrs	PA	**Intervention delivery** ▪ Intervention delivery over 4 alternative hours per week ▪ Intervention location walking distance from workplace **Intervention components** A 15-week PA intervention consisting of: ▪ Exercise training (60 minutes twice a week for 15 weeks) split into two groups: ▪ Aerobic capacity promoting training (music and Reebok steps; and international folk-dances) ▪ Strength exercises (circuit training: 12–15 repetitions and 2–3 series on each muscle group) ▪ Pulse rate watch used to measure PA intensity
McEachan et al (57)	UK	RCT	5 public workplaces (bus company; hospital; local govt council; national govt org; university)	Employees: Total N=1260 Intervention: N=662 Control: N=598 Gender: Male (%) Intervention: 45.2% Control: 46.8% Age: Intervention:43.1±10.4yrs Control: 42.5±10.8yrs	PA	**Intervention delivery** ▪ Delivered in the workplace ▪ Facilitators (1-5 per worksite) were volunteer employees with no prior specialist skills/knowledge but received 3-month training ▪ Facilitators free to choose different types of challenges depending on workforce **Intervention components** A 3-month PA intervention consisting of: ▪ Launch week (Week 1 of the intervention; facilitators were instructed to ‘launch’ the intervention, distribute the first of 3 interactive leaflets, display relevant posters, distribute self-monitoring fridge magnets and letters of management support, and run a ‘knowledge’ quiz) ▪ Team challenges ▪ Reminders ▪ Letters of management support ▪ Newsletters
Makrides et al (43)	Canada	RCT	8 employers in the greater Halifax area, Nova Scotia, Canada.	Employees: Total N=566 Intervention: N=282 Control: N=284 Gender (%) Men: Intervention: 53.4% Control: 50.7% Age: 44±8 yrs	Weight, PA, Diet (Smoking)	**Intervention delivery** ▪ Some employer support for participants to have flexible hours ▪ Health promotion program delivered at a variety of times **Intervention components** A 12-week health promotion program consisting of: ▪ Individual exercise prescriptions ▪ Supervised exercise classes ▪ Home exercise program ▪ Group education seminar ▪ Counselling ▪ Smoking cessation program ▪ Progress monitoring ▪ Discharge plan recommendations ▪ Telephone follow-up at 3 and 6 months post intervention
Williams et al (51)	USA	RCT	31 hotels on the island of Oahu, Hawaii	Employees: Total N=4536 Gender (%) Male:39.6% Female: 60.4% Age: Men=43.7±11.25yrs Women=45.5±11.24yrs	Weight, PA, Diet	**Intervention delivery** ▪ Two workplace employees designated as coordinators; tasks included scheduling activities, communicating with senior management about intervention and encouraging participation ▪ Employee coordinator’s time was paid **Intervention components** A 2-year weight loss and obesity prevention program consisting of: ▪ Raising employees’ awareness of their weight and health habits by providing feedback during their assessments ▪ Flyer about good health habits ▪ Group leaders ▪ Dietary education (the DASH diet) ▪ Environmental strategies: changes to cafeteria environments, wellness-themed contests and events, and increased stair use. ▪ Scrolling electronic signs, newsletters, flyers, posters, cafeteria table tents, and healthy choice stickers at the workplace to support healthy behaviors ▪ Promotion of healthier recipes, dishes, and portion sizes
Brox & Frøystein (60)	Norway	RCT	Community nursing home	Nurses and nurse aides: Total N=119 Intervention: N=63 Control: N=56 Gender: Women (%) Intervention: N=97% Control =96% Age: InterventioN=42.5yrs Control =42.5yrs	PA	**Intervention delivery** ▪ Exercise classes held weekly at two different times **Intervention components** A 6-month PA intervention consisting of: ▪ Fitness program: weekly 1 h session of light group exercise aerobic fitness ▪ Experienced instructors supervised exercise classes ▪ Classes regarding physical exercise, nutrition and stress management
Ribeiro et al (67)	Brazil	RCT	University hospital	Total: N=195 4 strand RCT with the following groups: Minimal treatment comparator group (MTC; N=7) Pedometer-based individual counselling group (PedIC; N=53) Pedometer-based group counselling (PedGC; N=48) Aerobic training group (AT; N=47 Gender: Women 100% Age: 40-50yrs	PA	**Intervention delivery** ▪ Interventions performed before or after working hours or during lunch period and on different days of the week **Intervention components** A 3-month four strand PA intervention consisting of: ▪ Minimal treatment comparator (MTP): 3 individual 15min sessions per month with researcher; given advice on PA (PA) benefits and booklet on PA; ▪ Pedometer-based individual counselling (PedIC): 3 individual 15min sessions per month with researcher; given advice on PA (PA) benefits, a booklet on PA, pedometer, diary to record total daily steps ▪ Pedometer-based group counselling (PedGC): 8 x 60 min group counselling session on PA benefits, overcoming barriers, self-monitoring (weekly for first 6 and last 2 sessions in 2-week interval) ▪ Aerobic training (AT): 24 sessions twice per week for 30-40 min) ▪ Health professionals (MTP, PedIC, PedGC) and experienced exercise professional (AT) facilitated sessions following prior training
Flannery et al (49)	USA	Quasi-experimental	Two long-term care facilities in Maryland	Female minority nursing assistants: Total N= 39 Intervention: N= 24 Control: N= 15 Gender: Female 100% Age: 42.39±12.79yrs	PA, Diet	**Intervention delivery** ▪ Intervention activities were conducted during paid work time ▪ Continuation of intervention activities after program completion was allowed and resources left to use (e.g. exercise DVDs) **Intervention components** A 12-week health promotion program consisting of: ▪ Environmental and policy assessment: workplace audit to assess factors that could influence healthy behaviors ▪ Education of Nursing Assistants (NAs): 1 x 30min group education lecture led by a nurse facilitator, using self-efficacy enhancement techniques ▪ On-going motivation: daily health tips; organized competitions; facilitated self-efficacy group discussions; nurse facilitator served as a resource person. ▪ Taste tests of healthy foods ▪ 3 x 10-min PA breaks each day were encouraged ▪ Group exercise classes (dance activities) ▪ Individualized goal setting & progress reports ▪ Pedometers ▪ Incentives (i.e. healthy groceries, small gift (e.g. lunch bag) given to participants who completed all measurements) ▪ Competitions ▪ Webpage ▪ Peer champions
Abood et al (50)	USA	Quasi-experimental (ex post facto research design)	A university campus worksite	University staff: Total N= 53 Intervention: N= 28 Control: N= 25 Gender: Female (%) Intervention: 96% Control: 92% Age: Intervention:34.3yrs Control: 37.9yrs	Diet	**Intervention delivery** ▪ Three education sessions were held each week to provide maximum opportunity for attendance ▪ Participants allowed 1 hour from workday to attend sessions **Intervention components** A 8-week nutrition education intervention consisting of: ▪ Weekly educational sessions: 8 x 1-hour sessions led by registered dietician ▪ Teaching combined with questions and answers, and information presented via computerized overhead projection, displays, and paper materials
Atlantis et al (12)	Australia	Pre and Post	An Australian casino	Employees: Total N=73 Gender: Female 52% Age: 32±8 yrs.	PA, Diet	**Intervention delivery** ▪ Timing of exercise sessions were not standardized owing to the varied work schedules ▪ Participants free to choose when to exercise between any of the available time periods **Intervention components** A 24-week exercise and lifestyle intervention consisting of: ▪ Supervised exercise prescription: supervised moderate-to- high intensity exercise including combined aerobic (at least 20 min duration 3 days/week) and weight-training (for an estimated 30 min 2–3 days/week) ▪ Behavior modification strategies: group seminars, one-on-one counselling (60 min/month per subject) and provision of a manual ▪ Incentives, e.g. ‘Bonus Activity Points’ awarded for compliance and redeemed for prizes (e.g. massage gift voucher)
Staley et al (44)	USA	Pre and Post	Four fire departments (54 stations total) located in central North Carolina	Fire fighters: Total N=190 Gender: Not specified Age: 40-55yrs	Physical activity	**Intervention delivery** ▪ All team competitions took place during the work day ▪ All necessary equipment was provided free of charge ▪ Participants and management co-produced the intervention ▪ Management support for allotted period for team competitions to take precedence over all nonemergency response activities **Intervention components** A 6-month PA intervention consisting of: ▪ Elements of the National Football League’s structure ▪ Competitions: Team-sport activities such as volleyball, basketball, flag football, or Frisbee football ▪ Participants involved in branding/naming the intervention ▪ Most physically fit team was recognized for best overall fitness outcome measures
Hess et al. (65)	Australia	Pre and Post	Liverpool Hospital	Employees: Total N= 339 Gender: MeN= 7.2% WomeN= 92.8% Age: 39.1± 10.9yrs	Physical activity, Diet	**Intervention delivery** ▪ Organizational changes put in place, including weekly walks for all staff (not limited to study participants) **Intervention components** A 12-week nutrition and PA intervention consisting of: ▪ Pedometer (record daily steps for 12 weeks) ▪ Healthy eating log book ▪ Weekly walks to complement intervention led by Health Promotion Staff ▪ Motivational and environmental strategies (posters with local walking routes and healthy messages; weekly motivational e-mails; ‘footprints’ directing people to use the stairs; and healthy messages on pay slips) ▪ Provision of information leaflet on process, water bottle; sandwich box; ‘healthy food fast’ cookbook and measure up campaign resources. ▪ Team challenges and prizes
Thorndike et al. (52)	USA	Pre and Post	Massachusetts General Hospital	Employees: Total N= 774 BMI<25: N= 277 BMI= 25–29.9: N= 250b BMI≥30: N= 230 Gender (%) Women BMI<25: 93% BMI= 25–29.9: 90% BMI≥30: 90% Age: BMI<25: 39±12.6 yrs BMI= 25–29.9: 42±11.2yrs BMI≥30: 44±10.8yrs	Physical activity, Diet	**Intervention delivery** ▪ Free provision of onsite health club ▪ No cost for participants; cost for employer ~$450 per person **Intervention components** A 10-week nutrition and PA intervention consisting of: ▪ Twice-weekly meetings; once as a whole group for a “rally” and a second time as 6 individual teams ▪ Team competitions for weight loss ▪ Goal-setting and relapse prevention ▪ Self-monitoring through logs of food intake, PA, and pedometer steps ▪ Free access to the onsite health club: weekly personal training and coupon for a healthy meal in the hospital cafeteria
Ferraro et al. (53)	USA	Pre and Post	A high-security prison service	Prison officers: Total N= 104 Gender (%) Men: 75% Women: 25% Age = 42.78±1.53 yrs	Weight, PA, Diet	**Intervention delivery** ▪ Intervention delivery team (DT) consisting of employees acting as the main voice for the program; responsible for implementation and recruitment ▪ The DT ensured scheduled weigh-ins covered all shifts **Intervention components** A 12-week weight-loss program with an 8-week weight-maintenance period (20 weeks in total) consisting of: ▪ Access to the educational material provided at intervention start with healthy eating guides and PA advice ▪ Bi-weekly bulletins with weight loss information posted in room dedicated to intervention participants (in workplace) ▪ Pedometer ▪ Raffle incentives based on achieving and maintaining individual weight loss goal
Giese et al (54)	USA	Pre and Post	Manufacturing plant	Diabetes prevention participants (enrolment criterion of BMI≥25): Total N=35 Gender: Females: 31 (89%) Males: 4 (11%) Age: Not specified	Weight	**Intervention delivery** ▪ The curriculum was offered in two time slots ▪ Curriculum offered at end of first and beginning of second shift ▪ Some employees could take time away from work and this was handled on an individual basis by manager (hourly employees attended on their own time) **Intervention components** A 16-week Diabetes Prevention Program Lifestyle Core Curriculum (publicly available online program) ▪ Curriculum offered in two time slots ▪ Fat and calorie reduction sessions offered by a company dietician ▪ Physical activity sessions offered by on-site fitness staff ▪ Behavioral change and mental health sessions facilitated on-site by clinical counsellor ▪ Nurse practitioner/certified diabetes educator facilitating all other sessions
Holtermann et al. (55)	Denmark	Study protocol for 3 RCTs and 1 Case-control exploratory study	Several workplaces in Denmark-cleaners, healthcare, construction, industrial workers	Predominant gender Cleaners: Female Healthcare: Female Construction: Male Industrial: Male	PA | Diet	**Intervention delivery** ▪ Information meeting conducted during working hours ▪ Intervention taking place during working hours (cleaners, construction); mainly during working hours (healthcare); at workplace and fitness center (industry) at own leisure time (employer covered fitness center costs) **Intervention components** ▪ Physical training: tailored to employee specific physical demands ▪ Cognitive behavioral theory based training (CBTr) ▪ Participatory ergonomics ▪ Diet
Jakobsen et al (56)	Denmark	Protocol RCT (single blinded cluster RCT)	Hospitals	Healthcare workers: Total N=200 Gender: female 100% Age: Exercise at work group: 40±12yrs Exercise at home group: 44±10yrs	PA	**Intervention delivery** ▪ Intervention activities during working hours in designated rooms located close to worksite departments **Intervention components** A 10-week physical activity intervention consisting of: ▪ 10 different forms of resistance training exercises ▪ 5 x 10 min exercise sessions per week ▪ Experienced instructors ▪ 5 group coaching sessions per individual (30-45min) ▪ Feedback to participants from instructors ▪ For ‘at home’ intervention group: bag with training equipment, posters demonstrating exercises ▪ Courses on ergonomic training
Geaney et al (62, 63)	Ireland	Protocol Cluster controlled	Manufacturing companies	Manufacturing workers Total: N=850 Total at follow-up: N=517 Gender: female 24% (at follow-up) Age groups (at follow-up): 18-29yrs: 8.5% 30-44yrs: 64% 45-65yrs: 27.5%	Diet	**Intervention delivery** ▪ Educational group sessions repeated a number of times per month so that all participants in all shifts have the opportunity to attend ▪ Each workplace had a research workplace leader based on-site for the duration of the study, to co-ordinate the study in collaboration with workplace stakeholders and monitor daily adherence to the interventions. **Intervention components** A 9-month four strand dietary intervention consisting of: ▪ Control: No changes implemented ▪ Nutrition education group: monthly group education sessions, individual nutrition consultations; healthy eating chat tables, detailed nutrition information via posters and leaflets, emails, menu labelling, quizzes, shopping cards, and personalized measurement cards. ▪ Environmental modification group: changes in workplace catering, including price discounts for fruit and vegetables, strategic positioning of healthier alternatives, portion size control, and restriction of fat/sugar/salt ▪ Nutrition education & Environmental modification group: combination of both groups
Sendall et al (45)	Australia	Pre and post	Transport industry	Truck drivers Total: N=44 Total at follow-up: N=22 Gender: male 100% Age at follow-up: Under 40yrs: 9 40yrs and older: 19	PA, Diet	**Intervention delivery** ▪ Intervention development used a Participatory Action Research (PAR) approach and was participant led ▪ Workplace managers decided which interventions to implement in their workplace based on capacity, logistical constraints, and assessment of perceived effectiveness of intervention in their workplace **Intervention components** ▪ A 6-month intervention consisting of three or four of the following health promotion interventions per worksite: ▪ Healthy eating posters displayed in workplace ▪ Healthy options in workplace vending machines ▪ Supply of free fruit to drivers ▪ A 10,000 step workplace challenge ▪ Healthy eating and/or physical activity toolbox talks at the workplace ▪ Health messages given to drivers, e.g. in their payslips ▪ A dedicated Facebook page (‘Truckin’ Healthy)
Naug et al (66)	Australia	Pre and post	Bus companies	Bus drivers Total: N=33 Gender: female 36% Age (average): 57yrs	Physical activity, Diet	**Intervention delivery** ▪ Intervention delivered in the workplace (i.e. depot training rooms) ▪ Participants were reminded of session times the previous day by text message **Intervention components** A 6-week intervention with a final session after another 6 weeks, consisting of: ▪ Three group education sessions around health education, physical activity and nutrition ▪ Session were designed to be interactive and fun and ended with pop-quiz game ▪ Pedometers

**Table 2:** Evidence Synthesis. [+++ Strong Evidence: consistent results in >2 studies of high quality; ++ Moderate evidence: consistent results in 1 high-quality study and 1 intermediate, or between some studies of intermediate quality; + Insufficient evidence: identification of only 1 study or inconsistent results across studies; - Evidence of no association: consistent results of a non-association in two or more studies.]

Outcomes of Interest					Other Outcomes			
Study	Quality Assessment	Weight	Healthy eating	Physical activity	Health^a^ (objective)	Health^b^ (self-reported)	Sickness absence	Work-related outcomes^c^
Morgan et al (64)	high							
Strijk et al (58, 68)	high							
Jakobsen et al (56, 69–71)	high							
Abood et al (50)	moderate							
Giese et al (54)	moderate							
Oldervoll et al (61)	moderate							
Thorndike et al (52)	moderate							
Atlantis et al (12)	moderate							
Brox & Frøystein (60)	moderate							
Ferraro et al (53)	moderate							
Flannery et al (49)	moderate							
Hess et al (65)	moderate							
McEachan et al (57)	moderate							
Pohjonen & Ranta (59)	moderate							
Ribeiro et al (67)	moderate							
Naug et al (66)	moderate							
Geaney et al (62, 63)	moderate							
Evidence synthesis		++	+	++	+	+	-	++
			Significant improvement		Non-significant change or inconsistent results			Significant negative effect

a Waist circumference, systolic/diastolic blood pressure, resting heart rate, VO2max, pain, total cholesterol, physical fitness, high-density lipoproteins.

b Self-perceived health status, feeling stressed/depressed.

c Work ability index, perceived work ability, need-for–recovery.

## Box

A systematic review was undertaken to identify the existing evidence for group-based interventions delivered within workplaces to help shift workers lose weight, increase physical activity, improve healthy eating, or reduce sedentary time. Our findings offer decision support on organisational-level adaptations and intervention components for the delivery of group-based workplace interventions that promote healthy lifestyles for shift workers.

## References

[1] Counterweight Project Team (2008). Influence of body mass index on prescribing costs and potential cost savings of a weight management programme in primary care. J Health Serv Res Policy.

[2] Gortmaker SL, Swinburn BA, Levy D, Carter R, Mabry PL, Finegood DT (2011). Changing the future of obesity: science, policy, and action. Lancet.

[3] Greener J, Douglas F, van Teijlingen E (2010). More of the same? Conflicting perspectives of obesity causation and intervention amongst overweight people, health professionals and policy makers. Soc Sci Med.

[4] King D (2011). The future challenge of obesity. Lancet.

[5] Wang YC, McPherson K, Marsh T, Gortmaker SL, Brown M (2011). Health and economic burden of the projected obesity trends in the USA and the UK. Lancet.

[6] U.S. Department of Health and Human Services (2003). Prevention Makes Common “Cents”. http://aspe.hhs.gov/health/prevention/.

[7] Goetzel RZ, Pei X, Tabrizi MJ, Henke RM, Kowlessar N, Nelson CF (2012). Ten modifiable health risk factors are linked to more than one-fifth of employer-employee health care spending. Health Aff (Millwood).

[8] Robroek SJ, Reeuwijk KG, Hillier FC, Bambra CL, van Rijn RM, Burdorf A (2013). The contribution of overweight, obesity, and lack of physical activity to exit from paid employment: a meta-analysis. Scand J Work Environ Health.

[9] National Institute for Health and Clinical Excellence (2008). Physical activity in the workplace (PH13). Public health guideline.

[10] Black C (2008). Working for a healthier tomorrow. Dame Carol Black’s Review of the health of Britain’s working age population.

[11] Medical Research Council (2010). A strategy for collaborative ageing research in the UK. Developed under the auspices of the Lifelong Health and Wellbeing Programme.

[12] Atlantis E, Chow CM, Kirby A, Fiatarone Singh MA (2006). Worksite intervention effects on physical health: a randomized controlled trial. Health Promot Int.

[13] Carpenter KM, Lovejoy JC, Lange JM, Hapgood JE, Zbikowski SM (2014). Outcomes and utilization of a low intensity workplace weight loss program. J Obes.

[14] DeJoy DM, Padilla HM, Wilson MG, Vandenberg RJ, Davis MA (2013). Worksite translation of the Diabetes Prevention Program: formative research and pilot study results from FUEL Your Life. Health Promot Pract.

[15] Faghri PD, Omokaro C, Parker C, Nichols E, Gustavesen S, Blozie E (2008). E-technology and pedometer walking program to increase physical activity at work. J Prim Prev.

[16] Goetzel RZ, Baker KM, Short ME, Pei X, Ozminkowski RJ, Wang S (2009). First-year results of an obesity prevention program at The Dow Chemical Company. J Occup Environ Med.

[17] Goetzel RZ, Roemer EC, Pei X, Short ME, Tabrizi MJ, Wilson MG (2010). Second-year results of an obesity prevention program at the Dow Chemical Company. J Occup Environ Med.

[18] Anderson LM, Quinn TA, Glanz K, Ramirez G, Kahwati LC, Johnson DB (2009). Task Force on Community Preventive Services. The effectiveness of worksite nutrition and physical activity interventions for controlling employee overweight and obesity: a systematic review. Am J Prev Med.

[19] Benedict MA, Arterburn D (2008). Worksite-based weight loss programs: a systematic review of recent literature. Am J Health Promot.

[20] Chapman LS (2004). Reducing obesity in work organizations. Am J Health Promot.

[21] Hutchinson AD, Wilson C (2012). Improving nutrition and physical activity in the workplace: a meta-analysis of intervention studies. Health Promot Int.

[22] Odeen M, Ihlebæk C, Indahl A, Wormgoor ME, Lie SA, Eriksen HR (2013). Effect of peer-based low back pain information and reassurance at the workplace on sick leave: a cluster randomized trial. J Occup Rehabil.

[23] Sorensen G, Stoddard A, Macario E (1998). Social support and readiness to make dietary changes. Health Educ Behav.

[24] Escoffery C, Kegler MC, Alcantara I, Wilson M, Glanz K (2011). A qualitative examination of the role of small, rural worksites in obesity prevention. Prev Chronic Dis.

[25] Neil-Sztramko SE, Pahwa M, Demers PA, Gotay CC (2014). Health-related interventions among night shift workers: a critical review of the literature. Scand J Work Environ Health.

[26] Proper KI, Koning M, van der Beek AJ, Hildebrandt VH, Bosscher RJ, van Mechelen W (2003). The effectiveness of worksite physical activity programs on physical activity, physical fitness, and health. Clin J Sport Med.

[27] Abraham C, Graham-Rowe E (2009). Are worksite interventions effective in increasing physical activity? A systematic review and meta-analysis. Health Psychol Rev.

[28] Ni Mhurchu C, Aston LM, Jebb SA (2010). Effects of worksite health promotion interventions on employee diets: a systematic review. BMC Public Health.

[29] Brown HE, Gilson ND, Burton NW, Brown WJ (2011). Does physical activity impact on presenteeism and other indicators of workplace well-being?. Sports Med.

[30] Cancelliere C, Cassidy JD, Ammendolia C, Cote P (2011). Are workplace health promotion programs effective at improving presenteeism in workers? a systematic review and best evidence synthesis of the literature. BMC Public Health.

[31] Jensen JD (2011). Can worksite nutritional interventions improve productivity and firm profitability? A literature review. Perspect Public Health.

[32] Odeen M, Magnussen LH, Maeland S, Larun L, Eriksen HR, Tveito TH (2013). Systematic review of active workplace interventions to reduce sickness absence. Occup Med (Lond).

[33] Papantoniou K, Vetter C, Schernhammer ES (2016). Shift work practices and opportunities for intervention. Occup Environ Med.

[34] Fujishiro K, Lividoti Hibert E, Schernhammer E, Rich-Edwards JW (2017). Shift work, job strain and changes in the body mass index among women: a prospective study. Occup Environ Med.

[35] Lindström J (2016). Does higher energy intake explain weight gain and increased metabolic risks among shift workers?. Scand J Work Environ Health.

[36] Hulsegge G, Boer JM, van der Beek AJ, Verschuren WM, Sluijs I, Vermeulen R (2016). Shift workers have a similar diet quality but higher energy intake than day workers. Scand J Work Environ Health.

[37] Kecklund G, Axelsson J (2016). Health consequences of shift work and insufficient sleep. BMJ.

[38] Ferri P, Guadi M, Marcheselli L, Balduzzi S, Magnani D, Di Lorenzo R (2016). The impact of shift work on the psychological and physical health of nurses in a general hospital: a comparison between rotating night shifts and day shifts. Risk Manag Healthc Policy.

[39] Harrington JM (2001). Health effects of shift work and extended hours of work. Occup Environ Med.

[40] Hall AL, Smit AN, Mistlberger RE, Landry GJ, Koehoorn M (2017). Organisational characteristics associated with shift work practices and potential opportunities for intervention: findings from a Canadian study. Occup Environ Med.

[41] Moher D, Schulz KF, Altman D, CONSORT Group (Consolidated Standards of Reporting Trials) (2001). The CONSORT statement: revised recommendations for improving the quality of reports of parallel-group randomized trials. JAMA.

[42] Des Jarlais DC, Lyles C, Crepaz N, TREND Group (2004). Improving the reporting quality of nonrandomized evaluations of behavioral and public health interventions: the TREND statement. Am J Public Health.

[43] Makrides L, Dagenais G, Chockalingam A, LeLorier J, Kishchuk N, Richard J (2008). Evaluation of a workplace health program to reduce coronary risk factors. Clin Govern Int J.

[44] Staley JA (2009). “Get Firefighters Moving”: Marketing a Physical Fitness Intervention to Reduce Sudden Cardiac Death Risk in Full-Time Firefighters. Soc Mar Q.

[45] Sendall MC, Crane PR, McCosker L, Biggs HC, Fleming ML, Rowland BD (2016). Workplace interventions to improve truck drivers’ health knowledge, behaviours and self-reported outcomes. Road Transp Res.

[46] Levac D, Colquhoun H, O’Brien KK (2010). Scoping studies: advancing the methodology. Implement Sci.

[47] Bernard B (1997). A critical review of epidemiologic evidence for work-related musculoskeletal disorders of the neck, upper extremity and low back.

[48] Steenstra IA, Verbeek JH, Heymans MW, Bongers PM (2005). Prognostic factors for duration of sick leave in patients sick listed with acute low back pain: a systematic review of the literature. Occup Environ Med.

[49] Flannery K, Resnick B, Galik E, Lipscomb J, McPhaul K, Shaughnessy M (2012). The Worksite Heart Health Improvement Project (WHHIP): feasibility and efficacy. Public Health Nurs.

[50] Abood DA, Black DR, Feral D (2003). Nutrition education worksite intervention for university staff: application of the health belief model. J Nutr Educ Behav.

[51] Williams AE, Vogt TM, Stevens VJ, Albright CA, Nigg CR, Meenan RT (2007). Work, Weight, and Wellness: the 3W Program: a worksite obesity prevention and intervention trial. Obesity (Silver Spring).

[52] Thorndike AN, Healey E, Sonnenberg L, Regan S (2011). Participation and cardiovascular risk reduction in a voluntary worksite nutrition and physical activity program. Prev Med.

[53] Ferraro L, Faghri PD, Henning R, Cherniack M, Center for the Promotion of Health in the New England Workplace Team (2013). Workplace-based participatory approach to weight loss for correctional employees. J Occup Environ Med.

[54] Giese KK, Cook PF (2014). Reducing obesity among employees of a manufacturing plant: translating the Diabetes Prevention Program to the workplace. Workplace Health Saf.

[55] Holtermann A, Jørgensen MB, Gram B, Christensen JR, Faber A, Overgaard K (2010). Worksite interventions for preventing physical deterioration among employees in job-groups with high physical work demands: background, design and conceptual model of FINALE. BMC Public Health.

[56] Jakobsen MD, Sundstrup E, Brandt M, Kristensen AZ, Jay K, Stelter R (2014). Effect of workplace- versus home-based physical exercise on pain in healthcare workers: study protocol for a single blinded cluster randomized controlled trial. BMC Musculoskelet Disord.

[57] McEachan RR, Lawton RJ, Jackson C, Conner M, Meads DM, West RM (2011). Testing a workplace physical activity intervention: a cluster randomized controlled trial. Int J Behav Nutr Phys Act.

[58] Strijk JE, Proper KI, van der Beek AJ, van Mechelen W (2009). The Vital@Work Study. The systematic development of a lifestyle intervention to improve older workers’ vitality and the design of a randomised controlled trial evaluating this intervention. BMC Public Health.

[59] Pohjonen T, Ranta R (2001). Effects of worksite physical exercise intervention on physical fitness, perceived health status, and work ability among home care workers: five-year follow-up. Prev Med.

[60] Brox JI, Frøystein O (2005). Health-related quality of life and sickness absence in community nursing home employees: randomized controlled trial of physical exercise. Occup Med (Lond).

[61] Oldervoll LM, Rø M, Zwart JA, Svebak S (2001). Comparison of two physical exercise programs for the early intervention of pain in the neck, shoulders and lower back in female hospital staff. J Rehabil Med.

[62] Geaney F, Kelly C, Di Marrazzo JS, Harrington JM, Fitzgerald AP, Greiner BA (2016). The effect of complex workplace dietary interventions on employees’ dietary intakes, nutrition knowledge and health status: a cluster controlled trial. Prev Med.

[63] Geaney F, Scotto Di Marrazzo J, Kelly C, Fitzgerald AP, Harrington JM, Kirby A (2013). The food choice at work study: effectiveness of complex workplace dietary interventions on dietary behaviours and diet-related disease risk - study protocol for a clustered controlled trial. Trials.

[64] Morgan PJ, Collins CE, Plotnikoff RC, Cook AT, Berthon B, Mitchell S (2011). Efficacy of a workplace-based weight loss program for overweight male shift workers: the Workplace POWER (Preventing Obesity Without Eating like a Rabbit) randomized controlled trial. Prev Med.

[65] Hess I, Borg J, Rissel C (2011). Workplace nutrition and physical activity promotion at Liverpool Hospital. Health Promot J Austr.

[66] Naug HL, Colson NJ, Kundur A, Santha Kumar A, Tucakovic L, Roberts M (2016). Occupational health and metabolic risk factors: A pilot intervention for transport workers. Int J Occup Med Environ Health.

[67] Ribeiro MA, Martins MA, Carvalho CR (2014). Interventions to increase physical activity in middle-age women at the workplace: a randomized controlled trial. Med Sci Sports Exerc.

[68] Strijk JE, Proper KI, van der Beek AJ, van Mechelen W (2012). A worksite vitality intervention to improve older workers’ lifestyle and vitality-related outcomes: results of a randomised controlled trial. J Epidemiol Community Health.

[69] Jakobsen MD, Sundstrup E, Brandt M, Andersen LL (2017). Psychosocial benefits of workplace physical exercise: cluster randomized controlled trial. BMC Public Health.

[70] Jakobsen MD, Sundstrup E, Brandt M, Jay K, Aagaard P, Andersen LL (2015). Effect of workplace- versus home-based physical exercise on musculoskeletal pain among healthcare workers: a cluster randomized controlled trial. Scand J Work Environ Health.

[71] Jakobsen MD, Sundstrup E, Brandt M, Jay K, Aagaard P, Andersen LL (2015). Physical exercise at the workplace prevents deterioration of work ability among healthcare workers: cluster randomized controlled trial. BMC Public Health.

[72] Barr-Anderson DJ, AuYoung M, Whitt-Glover MC, Glenn BA, Yancey AK (2011). Integration of short bouts of physical activity into organizational routine a systematic review of the literature. Am J Prev Med.

[73] Mozaffarian D, Afshin A, Benowitz NL, Bittner V, Daniels SR, Franch HA, American Heart Association Council on Epidemiology and Prevention, Council on Nutrition, Physical Activity and Metabolism, Council on Clinical Cardiology, Council on Cardiovascular Disease in the Young, Council on the Kidney in Cardiovasc (2012). Population approaches to improve diet, physical activity, and smoking habits: a scientific statement from the American Heart Association. Circulation.

[74] Kahn-Marshall JL, Gallant MP (2012). Making healthy behaviors the easy choice for employees: a review of the literature on environmental and policy changes in worksite health promotion. Health Educ Behav.

[75] Kaspin LC, Gorman KM, Miller RM (2013). Systematic review of employer-sponsored wellness strategies and their economic and health-related outcomes. Popul Health Manag.

[76] Holdsworth M, Haslam C, Raymond NT (2000). Does the heartbeat award scheme change employees’ dietary attitudes and knowledge?. Appetite.

[77] Hunt MK, Lederman R, Stoddard A, Potter S, Phillips J, Sorensen G (2000). Process tracking results from the Treatwell 5-a-Day Worksite Study. Am J Health Promot.

[78] Maes L, Van Cauwenberghe E, Van Lippevelde W, Spittaels H, De Pauw E, Oppert JM (2012). Effectiveness of workplace interventions in Europe promoting healthy eating: a systematic review. Eur J Public Health.

[79] Beresford SA, Thompson B, Feng Z, Christianson A, McLerran D, Patrick DL (2001). Seattle 5 a Day worksite program to increase fruit and vegetable consumption. Prev Med.

[80] Goetzel RZ, Ozmlnkowski RJ (2008). The health and cost benefits of work site health-promotion programs. Annu Rev Public Health.

[81] HSE (2015). Health and Safety Statistics, Annual report for Great Britain, 2014/2015. http://www.hse.gov.uk/statistics/overall/hssh1415.pdf.

[82] Nea FM, Pourshahidi LK, Kearney J, Livingstone MB, Bassul C, Corish CA (2017). A Qualitative Exploration of the Shift Work Experience: The Perceived Barriers and Facilitators to a Healthier Lifestyle and the Role of the Workplace Environment. J Occup Environ Med.

