# Supplementary material for: Group-based healthy lifestyle workplace interventions for shift workers: a systematic review
Source: Scand J Work Environ Health. Author manuscript; Available in PMC 2019 Aug 30. (PMC6716979; doi:10.5271/sjweh.3763)
Supplement: Supplementary Information [file EMS84161-supplement-Supplementary_Information.pdf]

SUPPLEMENTARY MATERIAL
Workplace lifestyle group-based interventions for shift workers

Table S1. Methodological quality appraisal of the included RCT studies (CONSORT Statement checklist).

		Morgan et al. 2011	Atlantis et al. 2006	Strijk et al. 2009	McEachan et al. 2011	Makrides et al. 2007	Brox and Frøystein, 2005	Ribeiro et al 2014	Jakobsen et al 2015	Jakobsen et al 2015	Jakobsen et al 2017
1a	Title	1	1	1	1	0	1	1	1	1	1
1b	Abstract	1	0.5	1	1	1	1	1	1	1	1
2a	Background	1	1	1	1	1	1	1	1	1	1
2b	Objectives	1	1	1	1	1	1	1	1	1	1
3a	Trial design	1	0.5	1	1	1	0.5	0.5	1	1	1
3b	Changes to trial design	0.5	0.5	0	0	0	0	0	0	0	0
4a	Participants	1	0.5	1	1	1	1	1	1	1	1
4b	Study settings	0.5	0.5	1	0.5	1	0.5	1	1	1	1
5	Interventions	1	0.5	1	1	1	1	0.5	1	1	1
6a	Outcomes	1	1	1	1	1	1	1	1	1	1
6b	Changes to outcomes	0.5	1	0	0.5	0	0.5	0	0	0	0
7a	Sample size	0.5	1	1	1	1	1	1	1	1	1
7b	Interim analyses and stopping guidelines	1	0.5	0	0.5	0	0.5	0	0	0	0
8a	Randomization: sequence generation	1	1	1	0.5	0	0.5	1	1	1	1
8b	Randomization: type	1	1	0.5	0	0	0	0.5	1	1	1
9	Randomization: allocation concealment mechanism	0.5	1	1	0.5	0	0	1	1	1	1
10	Randomization: implementation	1	0	0.5	0.5	0	1	0.5	0.5	0.5	0.5
11a	Blinding	1	0.5	1	1	0	1	0	1	1	1

11b	Similarity of interventions	0.5	0	0	0	0	0	0.5	0	0	0
12a	Statistical methods	1	1	1	1	1	1	1	1	1	1
12b	Additional analyses	1	1	1	1	0	0.5	0.5	1	1	1
13a	Participant Flow	1	1	1	1	1	1	1	1	1	1
13b	Losses and exclusions	0.75	1	1	1	1	1	1	1	1	1
14a	Dates defining the periods of recruitment and follow-up	1	1	1	1	0.5	1	1	1	1	1
14b	Reason for stopped trial	0	1	0	0.5	0	0.5	1	0	0	0
15	Baseline data	1	1	1	1	1	1	1	1	1	1
16	Numbers analysed	1	1	1	1	0.5	1	1	1	1	1
17a	Outcomes and estimation	1	1	1	0.5	1	1	1	1	1	1
17b	Binary outcomes	1	1	0	0	0	1	0.5	0	0	0
18	Ancillary analyses	0.5	1	1	1	0.5	0	0.5	1	1	1
19	Harms	0	0.5	1	1	0.5	0.5	0	0	1	0
20	Limitations	1	1	1	1	1	1	1	1	1	1
21	Generalisability (external validity)	1	1	1	1	0.5	1	1	1	1	1
22	Interpretation	1	1	1	1	1	1	1	1	1	1
23	Registration	1	0	1	1	0	0	1	1	1	1
24	Protocol	0	0	1	1	0	0	0	1	1	1
25	Funding	1	0	1	1	1	0	1	1	1	1
		37	37	37	37	37	37	37	37	37	37
		30.3	27.5	30.0	29.0	19.5	25.0	27.0	29.5	30.5	29.5
		82	74	81	78	53	68	73	80	82	80
		**high**	**moderate**	**high**	**moderate**	**low**	**moderate**	**moderate**	**high**	**high**	**high**

SUPPLEMENTARY MATERIAL
Workplace lifestyle group-based interventions for shift workers

Table S2. Methodological quality appraisal of the included non-RCT studies (TREND Statement checklist).

			Flannery et al. 2012	Abood et al. 2003	Staley et al 2009	Pohjonen and Ranta 2001	Oldervoll et al. 2001	Hess et al. 2011	Thorndike et al. 2010	Ferraro et al. 2013	Giese et al 2014	Sendall et al. 2016	Naug et al. 2016	Geaney et al. 2016
**1**	**Title and Abstract**	Information on how unit were allocated to interventions	0.5	1	0	0.5	0.5	0	0	0	0	0	0.5	1
		Structured abstract recommended	1	1	0	1	0	1	1	1	0	0	1	1
		Information on target population or study sample	1	1	0.5	1	1	1	0.5	1	1	1	1	1
**Introduction**														
**2**	**Background**	Scientific background and explanation of rationale	1	1	1	1	1	1	1	1	1	1	1	1
		Theories used in designing behavioral interventions	1	1	0.5	0.5	0.5	0.5	0	0.5	1	0	0.5	1
**Methods**														
**3**	**Participants**	Eligibility criteria for participants	1	1	0	1	1	1	1	1	1	1	1	1
		Method of recruitment	0.5	1	0	0.5	0.5	1	0.5	1	1	0.5	1	1
		Recruitment setting	1	1	1	1	1	1	1	1	1	1	1	1
		Settings and locations where the data were collected	1	0	0	0	0.5	1	1	1	1	1	1	1
**4**	**Interventions**	Details of the interventions intended for each study condition and how and when they were actually administered, specifically including:	0	0	0	0.5	0	0.5	0	0	0	1	1	1
		o Content	1	1	0.5	1	1	1	1	1	1	1	1	1
		o Delivery method	1	1	0.5	1	1	1	0.5	1	1	0.5	1	1
		o Unit of delivery	0.5	0.5	0.5	1	1	1	1	1	0	0.5	1	1

		o Deliverer	1	1	0	0.5	0.5	1	0	1	1	0	1	0.5
		o Setting	1	0.5	0.5	1	0.5	1	0.5	1	1	0.5	1	1
		o Exposure quantity and duration	1	1	1	1	0.5	1	0.5	1	1	0.5	1	1
		o Time span	1	1	0.5	1	1	1	1	1	1	1	1	1
		o Activities to increase compliance or adherence	1	0.5	0.5	1	1	1	0	0.5	0.5	0	1	0.5
**5**	**Objectives**	Specific objectives and hypotheses	1	0.5	0.5	1	1	1	0.5	1	1	0	1	1
**6**	**Outcomes**	Clearly defined primary and secondary outcome measures	1	0.5	0.5	1	0.5	1	1	1	1	0.5	1	1
		Methods used to collect data and any methods used to enhance the quality of measurements	1	1	0.5	1	1	1	0.5	1	1	0.5	1	1
		Information on validated instruments such as psychometric and biometric properties	1	0.5	0.5	1	1	1	0	1	1	0	0.5	1
**7**	**Sample Size**	How sample size was determined and, when applicable, explanation of any interim analyses and stopping rules	0	0	0	0	0	0	0	0.5	0	0	0	1
**8**	**Assignment Method**	Unit of assignment (the unit being assigned to study condition, e.g., individual, group, community)	1	1	0	1	1	1	1	1	1	0	0	1
		Method used to assign units to study conditions, including details of any restriction (e.g., blocking, stratification, minimization)	0.5	0	0	0.5	0	0	1	0	0	0	0	0
		Inclusion of aspects employed to help minimize potential bias induced due to non-randomization (e.g., matching)	0.5	0	0	1	0.5	0	0.5	0.5	0	0	0	0
**9**	**Blinding (masking)**	Whether or not participants, those administering the interventions, and those assessing the outcomes were blinded to study condition assignment; if so, statement regarding how the blinding was accomplished and how it was assessed.	0	0	0	0	0	0	0	0	0	0	0	0
**10**	**Unit of Analysis**	Description of the smallest unit that is being analysed to assess intervention effects (e.g., individual, group, or community)	0.5	1	0.5	1	1	0.5	1	1	0.5	1	1	1
		If the unit of analysis differs from the unit of assignment, the analytical method used to account for this (e.g., adjusting the standard error estimates by the design effect or using multilevel analysis)	0	0	0	NA	0	0	0	0	0	0	0	0

**11**	**Statistical Methods**	Statistical methods used to compare study groups for primary methods outcome(s), including complex methods of correlated data	1	1	0.5	1	1	1	1	1	1	0	1	1
		Statistical methods used for additional analyses, such as a subgroup analyses and adjusted analysis	1	1	0	1	1	1	1	1	0	0	0	0
		Methods for imputing missing data, if used	0.5	0	0	0.5	0	0	1	0	0	0	0	0
		Statistical software or programs used	1	0	0	1	1	1	1	1	0	0	0	1
**Results**														
**12**	**Participant flow**	Flow of participants through each stage of the study	0	0	0.5	1	1	0.5	1	0	0.5	1	0.5	1
		o Enrolment	1	0	0	0	1	0.5	1	0.5	1	0	0	1
		o Assignment	0.5	0.5	0	1	1	0.5	1	1	1	0	1	1
		o Allocation and intervention exposure	1	0.5	0	1	1	1	1	0.5	0.5	0	1	1
		o Follow-up	0.5	1	0	1	0.5	1	0.5	1	1	1	1	1
		o Analysis	0	0.5	0	1	1	0.5	0.5	1	1	1	1	1
		Description of protocol deviations from study as planned, along with reasons	0	0	0	NA	0	0	0	0	1	0	0	0
**13**	**Recruitment**	Dates defining the periods of recruitment and follow-up	0.5	0	0.5	0.5	0.5	0	0	0	0.5	0	0	0
**14**	**Baseline Data**	Baseline demographic and clinical characteristics of participants in each study condition	0.5	1	0	1	1	1	1	1	1	1	1	1
		Baseline characteristics for each study condition relevant to specific disease prevention research	0	0	0	1	0.5	0.5	1	0.5	0	1	1	1
		Baseline comparisons of those lost to follow-up and those retained, overall and by study condition	0	0	0	1	0	1	0	0	0	0	0	0
		Comparison between study population at baseline and target population of interest	0	0	0	0	0.5	0.5	1	0.5	0	0	0	1
**15**	**Baseline equivalence**	Data on study group equivalence at baseline and statistical methods used to control for baseline differences	0.5	1	0	0.5	1	0	1	0	0	0	0	1
**16**	**Numbers analysed**	Number of participants (denominator) included in each analysis for each study condition, particularly when the denominators change for different outcomes; statement of the results in absolute numbers when feasible	0	1	0	1	0.5	1	0.5	0.5	1	1	1	1

		Indication of whether the analysis strategy was "intention to treat" or, if not, description of how non-compliers were treated in the analyses	0.5	0.5	0	0	0	0.5	0.5	0	0	0	0	0.5
17	**Outcomes and estimation**	For each primary and secondary outcome, a summary of results for each estimation study condition, and the estimated effect size and a confidence interval to indicate the precision	0.5	1	0.5	1	1	1	1	1	1	0.5	0.5	0.5
		Inclusion of null and negative findings	1	1	0	1	1	1	1	1	1	0	1	1
		Inclusion of results from testing pre-specified causal pathways through which the intervention was intended to operate, if any	0.5	1	0.5	0.5	0.5	0.5	0	0.5	0	0	0	1
18	**Ancillary analyses**	Summary of other analyses performed, including subgroup or restricted analyses, indicating which are pre-specified or exploratory	0.5	1	0	1	0	0.5	0	0.5	1	0	0	0
19	**Adverse events**	Summary of all important adverse events or unintended effects in each study condition	0	0	0	0	0	1	0	0	0	0	0	0
**Discussion**														
20	**Interpretation**	Interpretation of the results, taking into account study hypotheses, sources of potential bias, imprecision of measures, multiplicative analyses, and other limitations or weaknesses of the study	1	1	0	1	1	1	0.5	1	1	0	1	1
		Discussion of results taking into account the mechanism by which the intervention was intended to work (causal pathways) or alternative mechanisms or explanations	0.5	1	0.5	0.5	0.5	0.5	1	1	1	0.5	0	1
		Discussion of the success of and barriers to implementing the intervention, fidelity of implementation	1	0.5	0.5	1	0.5	1	0	1	1	1	1	1
		Discussion of research, programmatic, or policy implications	1	1	0.5	1	0.5	1	1	1	1	1	1	1
21	**Generalizability**	Generalizability (external validity) of the trial findings, taking into account the study population, the characteristics of the intervention, length of follow-up, incentives, compliance rates, specific sites/settings involved in the study, and other contextual issues	0.5	0	0.5	0.5	1	0	0	0	0	1	1	1
22	**Overall Evidence**	General interpretation of the results in the context of current evidence and current theory	1	1	0.5	1	1	0.5	1	1	1	1	1	1
		Total items	59	59	59	57	59	59	59	59	59	59	59	59

Total score (%) and quality rate*	38 (64%) moderate	35.5 (60%) moderate	14.5 (25%) low	44 (77%) moderate	38 (64%) moderate	41 (69%) moderate	35.5 (60%) moderate	39.5 (67%) moderate	36.5 (62%) moderate	22.5 (38%) low	35.5 60%) moderate	46 (78%) moderate

* Study quality is rated as low, moderate or high if it scored less than 60%, between 60% and 79.9%, and 80% or more of the maximum score, respectively.
NA= not applicable

Table S3. Data extraction results from included studies

Outcome	Study ID	Results on associations between group-based interventions and primary (weight loss, healthy eating, BMI, Physical activity, health (objective and self-reported), and other outcome measures (sickness absence and work ability)
**Weight loss**	Morgan et al. 2011	• Significant effect for weight change at 14-week follow-up (P<.001; d=.34); mean difference between groups: 4.3 kg<br>• Significant difference in %-weight loss between groups (P<.001); mean %-weight loss in the Workplace Power (WP) group:−3.7% vs. +0.4% in control group<br>• At follow-up, significantly more WP participants (33.3%) lost more than 5% of baseline weight compared to the control group (0%) (χ^2=13.6, df=1, P<0.001)<br>• Significant treatment effects for BMI. Mean difference between treatment/control groups: 1.4kg/m2 (95%CI: 0.9, 2.0), p<0.001, ES= 0.41
	Giese et al 2014	• Statistically significant differences between weeks 1 and 16 in weight (Z = 3.89, p = <0.001, r =.66)<br>• Weight change ranged from a gain of 3.1% to a loss of 9.7% (median = 2.5% loss; IQR: 0.2% to 3.6% loss)<br>• Twenty-seven of the 35 participants lost weight: two had greater than 7% loss, five had 4%-7% loss, 12 had 2%-3% loss, two had 1% loss, and six <1% loss<br>• Eight participants gained weight: three had less than 1% gain and five had 1%-3.1% gain<br>• Significant differences between weeks 1 and 16 in BMI (Z = -3.83, p < .001, r = 0.65)
	Thorndike et al. 2010	• Mean overall weight loss: 1.9 kg (p<0.001) at the end of program; 0.4 kg (p=0.002) at 1 year follow<br>• Obese and overweight participants lost a significantly higher %-body-weight than normal weight participants at programme end<br>• No significant difference in weight loss between BMI categories at one-year follow-up
	Atlantis et al. 2006	• No significant change in weight loss between treatment (median: 0.1kg) and control group (median: 0.5kg); p=0.3<br>• No significant between group differences in changes in median BMI (p=0.3)
	Ferraro et al. 2013	• Mean weight loss at week 12: 10.01 lb [SE: 2.16] (p < 0.05)<br>• Baseline to week 20: intervention participants mean weight loss: 13.36 lb [SE 3.04] (p < 0.05), versus comparison group mean weight gained 4.72 lb [11.16] (non-significant)<br>• Significant reductions in BMI with 29% of participants moving from a higher BMI category to a lower category<br>• Participants lost a mean 1.35 kg/m2 [SE: 0.31] (p< 0.05) BMI at week 12;

		• Participants lost a mean 1.80 kg/m2 [SE: 0.40] ($p < 0.05$) of BMI at week 20; while the comparison group gained, on average, 0.43kg/m^2 [SE: 1.3140] - non-significant
	Ribeiro et al 2014	• Aerobic training (AT) was only intervention associated with a significant weight loss after 3 and 6 months (p = 0.05)<br>• 3-months weight change: MTC 0.239kg (95%CI: 0.1 to 0.3), PedIC -0.108kg (95%CI: -0.236 to 0.02); PedGC -0.192kg (95%CI: -0.750 to 0.366), AT -0.700kg (95%CI: -1.193 to 0.986)<br>• 6 months weight change: MTC 0.209kg (95%CI: 0.1 to 0.3), PedIC -0.136kg (95%CI: -0.23 to 0); PedGC -0.210kg (95%CI: -0.74 to 0.320), AT -0.740kg (95%CI: -1.23 to -0.25)
	Oldervoll et al. 2001	• No significant changes in BMI
	Flannery et al. 2012	• No treatment effect on BMI (p = 0.939)
	McEachan et al. 2011	• Negative intervention effect BMI- the intervention increased BMI by 0.18 units (95%CI: 0.01 to 0.34) compared to the control group
	Geaney et al. 2016	Compared to control group<br>• Nutrition education group: non-significant change in salt intake (p=0.144) versus control<br>• Environmental modification group: non-significant change in salt intake (p=0.459) versus control<br>• Nutrition education & Environmental modification group: Significant positive changes in dietary intakes of salt (−1.3 g/day (95% CI −2.3, −0.3), p = 0.010) between baseline and 7–9 months follow-up versus the control (fully adjusted model)<br><br>Change within each workplace at 7-9 month follow-up<br>• Nutrition education group: non-significant change in salt intake (−0.5 g/day (SD 4.1), p = 0.347)<br>• Environmental modification group: non-significant change in salt intake(−0.6 g/day (SD 5.5), p = 0.260)<br>• Nutrition education & Environmental modification group: Significant positive changes in dietary intakes of salt −1.4 g/day (SD 4.4), p = 0.000))
**Healthy Eating**	Morgan et al. 2011	• No treatment effect for fruit intake (mean change: 0.4serves/day (95%CI: −0.0, 0.8), p=0.06, Effect size (ES)=0.32, vegetables (mean change: 0.2serves/day (95%CI: −0.2, 0.6), p= 0.39, ES= 0.19), bread (mean change: −0.6 (95%CI: −1.3, 0.1), p= 0.09, ES= 0.38, milk (mean change: 1.3 (95%CI: −1.1, 1.8), p= 0.65, ES= 0.48), or diet drinks (mean change: 0.8 (95%CI:−0.3, 1.9), p= 0.17, ES= 0.27)<br>• There were intervention effects for cola (mean change: 1.2 (95%CI: 0.2, 2.1), p= 0.02, ES= 0.47) and soda/soft drink (mean change: 1.4 (95% CI: 0.4, 2.6), p=0.01, ES=0.60) but not for alcohol risk score which remained high (mean change: 0.3 (95%CI: −0.7, 1.2), p= 0.57, ES=0.10)<br>• Significant intervention effects for 'eating breakfast' and 'balancing food intake and physical activity'
	Abood et al. 2003	• Significant increase after treatment in perceived benefits of adoption of positive dietary behaviours; no significant change in perceived barriers to adoption of positive dietary behaviours<br>• Significant increase in nutrition knowledge related to CVD and cancer for treatment group (change pre-post: 4.56, p<0.001)<br>• Significant association between nutrition knowledge and higher fibre intake (p< .005), and between nutrition knowledge and consuming a lower percentage of total fat and saturated fat energy (p < .005)
	Strijk et al. 2012	• Intervention group workers significantly improved their fruit intake compared to the control group (+5.7 vs +2.7 pieces/week), resulting in an intervention effect on increasing fruit intake (β=2.7 pieces/week, 95%CI: 0.63 to 4.7)<br>• Stronger effects for fruit intake in the high compliance group of both the yoga (β=3.8 pieces, 95% CI 1.1 to 6.4) and the workout sessions (β=4.0pieces/week, 95% CI:1.1 to 6.4)
	Flannery et al. 2012	• No treatment effect for diet outcome expectations; significant positive time by treatment effect at 6 months (treatment 9.71, SD = 0.76,versus education only group 7.71, SD = 3.82, p =0.033)

		• Non-significant positive trends for time by treatment differences for average kilocalories (p = 0.062) at 3 months, favouring the treatment group<br>• No significant treatment effect for dietary fat and salt intake, but a trend toward significance observed for self-Efficacy for health related diet (p =0.069)
	Hess et al. 2011	• At completion, the daily requirement for fruit consumption was met by the majority of participants (81.8%) with a significant increase of 24.7% (p<0.001)<br>• Vegetable consumption was only met by a third of participants, however before-after change of 22.7% was significant (p<0.001)<br>• Significant changes in completers consuming breakfast seven days/week (change 16.6%, p<0.001) and consuming ≥1L of water (change 19.2%, p<0.001).<br>• Significant changes in people reporting they were on a diet (change 7.8%, p=0.007)<br>• Negative health behaviour outcome detected was a significant increase in participants (from 49.0% to 58.3%, p=0.008)) consuming one or more cups of a soft drink (including cordial or sports drinks)
	Naug et al. 2016	• Small but significant improvement in fruit consumption post-intervention<br>• Post-intervention 60% of participants reported adequate consumption of fruit (p=0.03)
	Sendall et al. 2016	• Consumption of the guideline-recommended number of daily serves of fruit increased by 23% (no indication if significant change)<br>• Consumption of the guideline-recommended number of daily serves of vegetables increased by 21% (no indication if significant change)
	Geaney et al. 2016	Compared to control group<br>• Nutrition education group: non-significant change in salt intake (p=0.144) versus control<br>• Environmental modification group: non-significant change in salt intake (p=0.459) versus control<br>• Nutrition education & Environmental modification group: Significant positive changes in dietary intakes of salt (−1.3 g/day (95% CI −2.3, −0.3), p = 0.010) between baseline and 7–9 months follow-up versus the control (fully adjusted model)<br><br>Change within each workplace at 7-9 month follow-up<br>• Nutrition education group: non-significant change in salt intake (−0.5 g/day (SD 4.1), p = 0.347)<br>• Environmental modification group: non-significant change in salt intake(−0.6 g/day (SD 5.5), p = 0.260)<br>• Nutrition education & Environmental modification group: Significant positive changes in dietary intakes of salt −1.4 g/day (SD 4.4), p = 0.000))
**Physical Activity**	Morgan et al. 2011	• Significant treatment effects for physical activity<br>• Mean change in Total MET minutes between groups: 0.3mins (95%CI: 0.0, 0.5), p=0.03, ES=0.77<br>• Mean change in Current PA level between groups: 0.6 (95%CI: 0.2, 1.0), p<0.001, ES=0.75<br>• Mean change in Workday PA between groups: 0.4 (95%CI: −0.2, 1.0), p= 0.18, ES=0.38<br>• Significant intervention effect for physical activity cons (mean difference between groups: 0.4 (95%CI: 0.1, 0.7), p= 0.01, ES=0.56) but not for physical activity pros (mean difference between groups: −0.1 (95%CI: −0.5, 0.2), p=0.48, ES=0.12) in physical activity cognitions
	Strijk et al. 2012	• Significant effectiveness on physical activity -i.e. sports activities (β: 40.4 min/week, 95% CI 13.0 to 67.7)<br>• No significant differences between groups in vigorous physical activity (VPA): (β: 48.5 min/week, 95% CI -81.0 to 178.1)<br>• No significant differences between groups in moderate-to-vigorous physical activity (MVPA): (SQUASH: β:1.4 min/week, 95% CI -126.0 to 123.2; CSA: β:13.8 min/week, 95% CI -25.9 to 53.5)
	Flannery et al. 2012	• No significant treatment effects for physical activity (average steps: p=0.9; average aerobic steps: p=0.259; average aerobic mins: p=0.242; average kilocalories: p=0.866)<br>• Significant positive time by treatment effect at 3 months for daily average of aerobic mins (treatment 9.59 mins, SD = 12.77, education only 6.00 mins, SD = 16.49, p = 0.05)<br>• Non-significant positive trends for time by treatment differences for average aerobic steps (p = 0.058) and average kilocalories (p = 0.062) at 3 months, favouring the treatment group

	Hess et al. 2011	• Significant increase across all physical activity measures among initially inactive participants (performing <150 min physical activity per week). Significant before-after changes in: Number of times spent walking 10 min or more: $p<0.001$; Minutes spent walking past week: $p<0.001$; Minutes spent doing moderate PA last week: $p<0.001$; Minutes spent doing vigorous PA last week: $p<0.001$<br>• Smaller increase in initially active participants (≥150min physical activity per week). In this group, moderate physical activity did not increase significantly (p=0.43); the increase in vigorous physical activity was just significant (p=0.045)
	McEachan et al. 2011	• Non-significant effect on self-reported moderate/vigorous physical activity, controlling for past physical activity (β = 52.70, 95%CI: -132.92-38.32; p=0.578)
	Pohjonen and Ranta 2001	• Frequency of leisure-time physical activity did not differ statistically significantly between the baseline and the 5-year follow-up in either the intervention or the control group.<br>• Subjects in the intervention (67%) and control groups (40%) categorized as highly physically active before the intervention also exercised at high level after the 5-year follow-up
	Ribeiro et al 2014	• Individual pedometer-based individual counselling (PedIC) and group counselling (PedGC) significantly increased the total number<br>• of steps after 3 months (512 and 1475 steps per day, respectively) compared with the minimal treatment comparator (MTC) (-597 steps per day, p< 0.05)<br>• Significant increase in number of steps at moderate intensity was also observed in the PedGC group after 3 months when compared with the PedIC group and MTC (PedGC, 845 steps per day, p<0.05).<br>• No significant change in the total number of steps after 3 months from the AT group (p> 0.05)<br>• No significant change in total and moderate steps for any intervention group after 6 months
	Naug et al. 2016	• All participants that completed pot-intervention questionnaire (N=16) reported an increase in exercise levels.<br>• No indication if change was significant or not.
	Sendall et al. 2016	• No improvement in physical activity behaviours<br>• Positive changes in sedentary behaviour (no indication if significant or not). Number of drivers reporting sitting 9 or more hours per day decreased by 26%.
**Health (Objective)**	Morgan et al. 2011	• Significant treatment effects for waist circumference (mean difference between groups: 5.9cm (95%CI: 4.2, 7.6), p<0.001, ES=0.63), systolic blood pressure (mean difference between groups: 6.0mmHg (95%CI: 0.8, 11.2), p=0.02, ES=0.48), resting heart rate (mean difference between groups: 7.9bpm (95%CI: 4.0, 11.7), p<0.001, ES=0.81)<br>• Medium-to-large effect sizes (range from d=.41–.81) were found<br>• No treatment effect was found for diastolic blood pressure (mean difference between groups: 1.2mmHg (95%CI: −2.4, 4.7), p=0.52, ES=0.18)
	Strijk et al. 2012	• No significant effects were found on aerobic capacity (VO_{2max}) (β=0.231(95%CI: 0.82-1.03) or mental health (β=01.04(95%CI: -0.70-2.78)
	Oldervoll et al. 2001	• No overall significant group difference in VO_{2max} (F(2/39) = 0.43), and no significant overall change of VO_{2max} from pre- to post-test (F(1/39) = 0.77) after 15 weeks<br>• No significant difference in pain $index_{pre}$ scores (F(2/42) = 1.31) after 15 weeks<br>• At 7 month follow-up for the pain index, ET-group increase their pain score from 4.1 at post-test to 5.7, but were significantly better than before intervention (t = 2.08, p = 0.05); the SP-group increased their score from 5.3 to 6.8 at follow-up and reported significantly less pain than before intervention (t = 5.32, p = 0.0001); the CON-group reduced mean pain score from 11.0 at post to 6.8 at follow-up and pain levels were on average significantly better than before intervention (t = 2.44, p = 0.03)
	Thorndike et al. 2010	• Waist circumference (change -3.6cm; p<0.001), systolic blood pressure (change -2.6mmHg, p<0.001), diastolic pressure (change -1.9, p<0.001), and total cholesterol (change -7.7mg/dL, p<0.001) significantly decreased for all participants at the end of the program<br>• At 1 year follow-up, significant changes observed for waist circumference (change -1.6cm; p<0.001), diastolic pressure (change -1.5, p<0.001), and total cholesterol (change -1.9mg/dL, p=0.002), but non-significant change for systolic blood pressure (change -0.4mmHg, p=0.30)

	Atlantis et al. 2006	• At 24 weeks, significant between-group differences observed , after adjusting for baseline values, for predicted VO2max (43.4±10.1 versus 40.9±10.2 ml/kg/min, p= 0.02), favouring the intervention<br>• An additional 1000 weight-training repetitions predicted an increase in the mean predicted VO_{2max} measure of 1 ml/kg/min (95% CI:0–1ml/kg/min, r^2=20%, p=0.003)
	Brox and Frøystein, 2005	• No significant change in physical fitness (mean difference between groups: 0.1 (95%CI: 0.3-0.4), p=0.92) and overall health (mean difference between groups: 0.0 (95%CI: 0.3-0.3), p=0.66)
	Flannery et al. 2012	• Significant positive treatment effects for total cholesterol (p=0.002) and triglycerides (p=0.011)<br>• Significant negative treatment effect for high-density lipoproteins (HDLs; p≤0 .001)<br>• Significant positive treatment effect for average systolic BP (p=0.028)<br>• No significant treatment effect for diastolic BP, but a positive trend for significance observed (p=0.073)<br>• No significant treatment effect on low-density lipoproteins observed (LDLs; p=0.635)<br>• Significant positive treatment effect for depressive symptoms (p=0.012)
	McEachan et al. 2011	• No significant effect of the intervention on diastolic blood pressure or percentage body fat (results not reported)<br>• Significant effects of the intervention on systolic blood pressure (difference between groups:-1.79 mmHg; 95%CI = -3.10 to -0.47), resting heart rate (intervention group 2.08 beats less than control (95%CI: -3.28 to -.089)
	Pohjonen and Ranta 2001	• Absolute VO_{2max} and VO_{2max} in relation to body weight were improved an average of 5%, and 7%, respectively at the 1-year follow-up. In the covariance analysis, the least- squares means of the changes of above variables were statistically significant in the intervention group (p<0.001 and p=0.014, respectively).The difference were not significant at the 5-year follow-up (p=0.339 and p=0.650, respectively)
	Naug et al. 2016	• Of all the participants that completed the program (N=21), 28% improved their risk status, with improvements in blood pressure, HDL-C (Δ_{HDL-C}=0.19; p=0.003); waist circumference and fasting glucose ($\Delta_{glucose}$=0.11; p=0.580)
	Geaney et al. 2016	Change compared to control at 7-9 month follow-up<br>• Nutrition education group: non-significant change in BMI (p=0.196) versus control<br>• Environmental modification group: non-significant change in BMI (p=0.711) versus control<br>• Nutrition education & Environmental modification group: Significant changes in BMI (−1.2 kg/m2 (95% CI −2.4, −0.1), p=0.047) versus control<br><br>Change within each workplace at 7-9 month follow-up<br>• Nutrition education group: small significant change in BMI (−0.2 kg/m2 (SD 1.0), p = 0.009)<br>• Environmental modification group: non-significant change in BMI (−0.1 kg/m2 (SD 1.0), p =0.590)<br>• Nutrition education & Environmental modification group: Significant positive changes in BMI (−0.3 kg/m2 (SD 0.8), p =0.001)
**Health Subjective**	Pohjonen and Ranta 2001	• Significant improvement in perceived health status between the groups differed during the first follow-up period; 71% of intervention group improved from the poor to the good health status category after the physical exercise intervention (p=0.003), whereas in the control group the corresponding proportion was 47% (p=0.125)
	Hess et al. 2011	• Significant change in proportion feeling stressed all the time or most of the time (p=0.003)<br>• No significant change in proportion feeling depressed all the time or most of the time (p=0.08)
	Brox and Frøystein, 2005	• No significant change in self-perceived change in health status (mean difference between groups: 0.2 (95%CI: 0.1- 0.6), p=0.19)

	Sendall et al. 2016	• Improvement in drivers' self-reported health ratings, with the number of drivers reporting their health as 'good', 'very good', or 'excellent' increasing by 11% (no indication if change was significant or not)<br>• Improvements in self-reported BMI, with the number of drivers reporting their BMI as 'obese' decreasing by 16% (no indication if change was significant or not)
	Jakobsen et al 2015a, 2015b, 2017	• Group by time interactions were observed for vitality, control and concern about pain (p < 0.05) which corresponded to small effect sizes (Cohen d = 0.27–0.36) in favor of workplace-based physical exercise.<br>• Mental health remained unaltered<br>• A group-time interaction was reported for pain intensity (P=0.01). Compared with HOME, average pain intensity decreased [-0.7 (95% CI -1.0 to -0.3)] in the WORK group and a similar pattern was observed for regional lower-back pain (P=0.02) whereas no change was seen for the neck/shoulder (P=0.09) pain intensity.<br>• A group-time interaction was observed for lower-back muscle strength (P<0.001). Compared to HOME, lower-back muscle strength increased to a greater extent in the WORK group<br>• For WORK participants, 78% experienced some or much improvement of pain, while similar changes were seen in 42% of the pain-case HOME participants (P=0.006)<br>• Effect size for the change in pain was 0.31, which was categorized as small (from 0.20 to 0.50).
**Sickness Absence**	Brox and Frøystein, 2005	• Sickness absence increased in both the intervention and control groups although the increase was significant only in the intervention group (P= 0.03)<br>• Non-significant between groups difference in total days of sickness absence (difference between groups: 4.7 days (95%CI:5.7 to 15.0), p=0.64) in favour of the control group<br>• No change for self-certified sick days
	Jakobsen et al. 2015a, 2015b, 2017	• Of the seven items of WAI, item 2 (work ability in relation to the demands of the job) and item 5 (sickness absence during the last year (12 months)) increased following WORK compared with HOME (p < 0.05; Table 2).<br>•
**Work Ability**	Pohjonen and Ranta 2001	• The index and the perceived estimation of current work ability were similar at the baseline level the 1-year follow-up, among the subjects who participated in the exercise intervention<br>• In the control group the decline of the work ability index was about three times faster than in the intervention group during the 5-year period<br>• In the intervention group, the average value of the index remained "good" from the baseline (42.2 (SD 3.2) points) to the 5-year follow-up (40.6 (SD 4.5) points) measurements. Respectively, in the control group during the 5-year period, the mean value of the index decreased from 37.7 (SD 6.1) to 33.8 (SD 8.3) and the corresponding work ability category declined from "good" to "moderate."<br>• The subjective estimation of the current work ability was, on average, 8.7 (SD 0.8) at the baseline and 8.2 (SD 0.7) after the 5-year follow-up in the intervention group and 7.9 (SD 1.3) versus 6.6 (SD 1.9) in the control group
	Strijk	• For the work-related outcome of need-for-recovery (NFR), the intervention group significantly decreased their NFR more compared to the control group (-3.2 vs 0.6).<br>• The intervention effectively decreased NFR (β=-3.5 points, 95% CI:-6.4 to -.54, p<0.05)
	Jakobsen et al. 2015a, 2015b, 2017	• Work pace increased more in WORK intervention group compared with HOME (p < 0.05)<br>• Work disability, emotional demands, influence at work, sense of community and social support from managers remained unaltered<br>• A group by time interaction for work-ability index (WAI)(p = 0.03) was reported.<br>• WAI increased in WORK compared with HOME (Δ_{WAI}=1.1 (0.3, 1.8; p=0.03)

		• No within-group change in WAI was observed for participants in WORK (p = 0.52) whereas WAI decreased (i.e. worsened) in HOME (p = 0.02).<br>• Of the seven items of WAI, item 2 (work ability in relation to the demands of the job) and item 5 (sickness absence during the last year (12 months)) increased following WORK compared with HOME ($p < 0.05$; Table 2).<br>• No changes in the remainder WAI items were observed.<br>• Effect size (Cohen's d) of the change in WAI score with WORK compared with HOME was 0.24, which was categorized as small-to-moderate (0.20 to 0.50).
